# Antibiotic Treatment Reduced the Gut Microbiota Diversity, Prolonged the Larval Development Period and Lessened Adult Fecundity of *Grapholita molesta* (Lepidoptera: Tortricidae)

**DOI:** 10.3390/insects13090838

**Published:** 2022-09-15

**Authors:** Xuan Zhang, Xing Wang, Zikun Guo, Xueying Liu, Ping Wang, Xiangqun Yuan, Yiping Li

**Affiliations:** 1 Key Laboratory of Integrated Pest Management on Crops in Northwestern Loess Plateau, Ministry of Agriculture, College of Plant Protection, Northwest A&F University, Yangling, Xianyang 712100, China; 2 Key Laboratory of Plant Protection Resources and Pest Management, Ministry of Education, College of Plant Protection, Northwest A&F University, Yangling, Xianyang 712100, China; 3 Department of Entomology, Cornell University, Ithaca, NY 14850, USA

**Keywords:** oriental fruit moth, 16S rDNA, ciprofloxacin, oviposition, culturable bacteria

## Abstract

**Simple Summary:**

The gut microbiota, which has an important influence on insect physiology and ecology, can be affected by many factors, such as antibiotics, temperature, diet, and species. However, herein we demonstrate the effects of antibiotics on the gut microbiota and on *Grapholita molesta* development and fecundity. Five antibiotics, ciprofloxacin, streptomycin, chloramphenicol, ampicillin, and rifampicin, were screened by the drug-sensitive disc method. Then, the effects of antibiotics on oriental fruit moth growth, reproduction, and gut microbiota structure were investigated by comparison of biological parameters and 16S amplicon sequencing technology. The results illustrated ciprofloxacin, showing the largest inhibition zone diameter, to be the most suitable antibiotic to inhibit the gut microbiota of *G. molesta* a minimum inhibitory concentration of 1 μg/mL. After feeding ciprofloxacin, the relative abundance of Actinobacteria and Cyanobacteria significantly decreased, while that of Bacteroidetes and Firmicutes increased. Finally, ciprofloxacin feeding affected larval growth, development, and reproduction significantly, resulting in prolonged larval development duration, shortened adult longevity, and significantly decreased single female oviposition and egg hatchability. In addition, we isolated and purified some culturable bacteria belonging to Proteobacteria, Firmicutes, Actinobacteria, and cellulase-producing bacteria from the *G. molesta* midgut.

**Abstract:**

*Grapholita molesta*, the oriental fruit moth, is a serious pest of fruit trees with host transfer characteristics worldwide. The gut microbiota, which plays a crucial part in insect physiology and ecology, can be influenced by many elements, such as antibiotics, temperature, diet, and species. However, the effects of antibiotics on *G. molesta* gut microbiota are still unclear. In this study, we selected five common antibiotic agents to test the inhibition of *G. molesta* gut microbiota, and found ciprofloxacin shown the best antibacterial activity. After feeding 1 μg/mL of ciprofloxacin, the relative abundance of Actinobacteria and Cyanobacteria decreased significantly, while that of Firmicutes and Bacteroidetes increased. PICRUSt2 analysis indicated that most functional prediction categories were enriched in the *G. molesta* gut, including amino acid transport and metabolism, translation, ribosomal structure and biogenesis, carbohydrate transport and metabolism, transcription, cell wall/membrane/envelope biogenesis, and energy production and conversion. Finally, ciprofloxacin feeding significantly affected larval growth, development, and reproduction, resulting in prolonged larval development duration, shortened adult longevity, and significantly decreased single female oviposition and egg hatchability. In addition, we isolated and purified some culturable bacteria belonging to Proteobacteria, Firmicutes, Actinobacteria, and cellulase-producing bacteria from the *G. molesta* midgut. In brief, our results demonstrate that antibiotics can have an impact on *G. molesta* gut bacterial communities, which is beneficial for host growth and development, as well as helping female adults produce more fertile eggs. These results will thus provide a theoretical reference for developing new green control technology for *G. molesta*.

## 1. Introduction

The oriental fruit moth, *Grapholita molesta* (Busck), is a pest of fruit trees that seriously affects agricultural economic development in most temperate regions of the world [1,2,3]. The first and second generations of larvae bore into the tender twigs of peach, plum, cherry, and other fruit trees in the family Rosaceae in spring and early summer, causing shoot dieback [4]. Later generations directly burrow into the fruit, turning it black and causing it to rot or fall off [5,6]. At present, the foremost methods of controlling *G. molesta* rely on the application of insecticides during the egg and larval stages and the use of sex pheromones specifically designed for male moths in the adult stages [3,7]. However, due to the biological characteristics of *G. molesta* in concealing damage, host switching, and generation overlapping, the pesticides do not achieve the desired effect [2,8]. In addition, with the widespread use of chemical pesticides, the development of resistance in *G. molesta* to multiple insecticides has generally increased [9].

Previous studies have indicated that an in-depth study of insect symbiotic microorganisms, especially diet-related gut microbiota, is essential for understanding the biological and ecological characteristics of the host and may provide a new entry point for integrated pest management [10,11]. Gut bacteria play an indispensable role in the long-term coevolution of insects and symbiotic microbes and microorganisms, especially in Lepidoptera. They directly or indirectly play a role in host digestion and absorption [12,13,14], immune regulation [15,16], pesticide metabolism [17,18,19], mating and reproduction [20], information transmission, and other life activities [21,22], while the host also affects intestinal microbes in morphology, physiology, and ecology, promoting the coevolution of intestinal microbes [23].

A large number of studies have confirmed that the diversity and homeostasis of insect gut microbiota are influenced by many factors, such as temperature, weather, diet, and species [21,23,24,25], among which diet and species are the two most important factors [10,26,27,28,29,30]. Antibiotic treatment has been widely used to research the role of insect gut microbiota or target bacteria [24,31,32,33,34,35]. Five successive generations of *Laodelphax striatellus* (SBPH) was raised by adding 1 g/L tetracycline to rice, the numbers of Firmicutes, Bacteroidetes, Tenericutes, and Fusobacteria in the gut of the *L. striatellus* were significantly reduced, and *Wolbachia, Bacteroides*, and *Abiotrophia* were almost 100% eliminated [25]. The mating selectivity of *Drosophila melanogelis* disappeared on both honey medium and starch medium separately after the intestinal bacteria were removed [22]. Further studies found that the gut microbiota could influence mating preference by changing the level of hydrocarbon pheromone in the epidermis of *D. melanogelis* [22]. After the first-instar larvae of *Zeugodacus tau* were fed antibiotics, the bacterial diversity of the adult stage changed significantly. Untreated flies can lay eggs normally, while the development of the ovary of the treated flies is inhibited and unable to lay eggs, which may be related to the change of microbiota [36]. After tetracycline treatment, the diversity of symbiotic bacteria in *Nilaparvata lugens* decreased, the relative content of *Wolbachia* decreased significantly, the expression level of the *NlCYP4CE1* gene was significantly downregulated, the activity of cytochrome P450 decreased significantly, and the metabolic capacity of imidacloprid decreased significantly. These results indicated that *Wolbachia* could promote the expression of the *NlCYP4CE1* gene in *N. lugens*, thus promoting imidacloprid metabolism [19].

In a previous study, we demonstrated that the structure of the *G. molesta* gut microbiota is variable in response to different host plants [37]. However, the role of gut microbiota in the growth and development of *G. molesta* is still unknown, and the effects of antibiotic feeding on the structure of gut microbiota and growth and development of *G. molesta* have not been reported. Therefore, this study aimed to reveal the effects of antibiotic feeding on the structure of intestinal microbiota and the growth and reproduction of *G. molesta*, so as to explore the important role of gut microbiota in the growth, development, and reproduction of *G. molesta*.

## 2. Materials and Methods

### 2.1. Insect Reared and Sample Processing

The *Grapholita molesta* used in this experiment were obtained from a population that had been artificially reared for six years at 26 ± 0.5 °C, 70 ± 10% relative humidity (RH), and 15 h:9 h photoperiod (L:D) in the Key Laboratory of Plant Protection Resources and Pest Management, Ministry of Education, College of Plant Protection, Northwest A&F University, Yangling, Shaanxi, China. Artificial feed consisted of corn flour 107.14 g/L, soybean flour 107.14 g/L, yeast 42.86 g/L, ketchup 282.86 g/L (TONG QIAN QIAO, Ningbo Tongqianqiao Food Development Co. Ltd. Ningbo, China), ascorbic acid 4.29 g/L, cholesterol 0.14 g/L, sorbic acid 1.43 g/L, methylparaben 2.86 g/L, and agar powder 20 g/L. Twenty 4th-instar larvae were surface-sterilized in 75% ethanol for 60 s and rinsed three times with sterile water for 30 s. Midgut tissues were dissected in a 90 mm diameter sterile petri dish under a SE 2200 insect stereoscope (Germany Zeiss Optics International Group Co., Ltd. Oberkochen, Germany.) with sterile forceps and rinsed with sterile phosphate-buffered saline (PBS), then removed to a sterile centrifuge tube containing 1000 μL of PBS. The midgut was homogenized under a sterilized environment for preparation.

### 2.2. Screening of Types and Concentrations of Antibiotics

The sensitivity of intestinal microbiota to antibiotics was determined by the drug-sensitive paper method [38], and five antibiotics, including ampicillin, streptomycin, rifampicin, ciprofloxacin, and chloramphenicol, were used for the study [36,39,40]. On a SW-CJ-2D clean bench (Suzhou Purifying Equipment Co., Ltd. Suzhou, China), 100 μL of the grinding solution in Section 2.1 was diluted 10 times, 50 μL of diluent was evenly spread on the Mueller–Hinton Broth (MH, Qingdao Haibo Biotechnology Co., Ltd. Qingdao, China) agar plates (beef extract 2.0 g/L, casamino acid 17.5 g/L, soluble starch 1.5 g/L, agar 15 g/L), and then the drug-sensitive paper standard (each tablet contains only one antibiotic, ciprofloxacin 5 μg/tablet, streptomycin 10 μg/tablet, chloramphenicol 30 μg/tablet, rifampicin 5 μg/tablet, and ampicillin 10 μg/tablet, Hunan BKMAM Biotechnology Co., Ltd. Hunan, China) was stuck onto the surface of the plates, pressing it firmly with tweezers. According to the pre-experiment, the antibiotic with a large antibacterial circle would be 1 piece per plate, and the antibiotic with a small antibacterial circle would be three pieces per plate. After sealing the plate, it was placed upside down in an incubator at 30 °C, for 24 h. The diameter of the antibacterial circle was measured by drawing a circle around the antibacterial circle at the bottom of the petri dish and recorded.

The antibiotic with the best bacteriostatic effect was selected, and the optimal bacteriostatic concentration was explored by the microbroth method according to the CLSI (2012) standard [38]. Then, 100 μL of the grinding solution prepared in Section 2.1 was added into 50 mL of MH liquid medium, the medium was placed in a full-temperature oscillation incubator at 37 °C and 150 r/min for 6 h for enrichment, and the enriched bacterial solution was diluted 1000 times as the sample bacterial solution. A 0.512 mg/mL antibiotic ciprofloxacin (Shanghai Macklin Biochemical Co., Ltd. Shanghai, China) solution was prepared, and using 11 1.5 mL sterile centrifuge tubes, 200 μL of MH liquid medium was added into each tube. Then, 200 μL of ciprofloxacin solution was added to Tube 1, and a serial dilution curve of ciprofloxacin was made from Tube 1 to 11, obtaining the following final concentrations of 256, 128, 64, 32, 16, 8, 4, 2, 1, 0.5, and 0.25 μg/mL. Then, 200 μL of sample bacterial solution was added to each tube, and the final concentrations of ciprofloxacin were 128, 64, 32, 16, 8, 4, 2, 1, 0.5, 0.25, and 0.125 μg/mL, respectively. In addition, only 400 μL of sample bacterial solution without antibiotics was added as a positive control, and only 400 μL of MH liquid medium was added as a negative control. To prove that water as a solvent will not have a significant effect on the experiment, 200 μL of ddH_2_O and 200 μL of sample bacterial solution were added as blank controls. Then, the control group and the treatment group were simultaneously placed in a full-temperature oscillation incubator at 37 °C and 150 r/min for 24 h, and the *OD*_600_ value was determined with a Synergy HTX microplate analyzer (Gene Company Limited, Hong Kong, China). Each group had 3 replicates. Statistical analysis software SPSS 26.0 was used to calculate the diameter of the bacteriostatic zone of various antibiotics and *OD*_600_. One-way analysis of variance (ANOVA) and Tukey post-hoc test were used for analyzing the difference of the measured values at *p* < 0.05 (similarly hereinafter).

### 2.3. Antibiotic Treatment

According to the results in Section 2.2, 1 μg/mL was the minimum inhibitory concentration of ciprofloxacin. *G. molesta* eggs produced on the same day were fed with an artificial diet containing 1 μg/mL of ciprofloxacin, and the control group was fed the artificial diet without antibiotics. Feeding conditions and artificial diet formulations are shown in Section 2.1. The larvae were reared to the 4th instar for 16S rDNA sequencing.

### 2.4. DNA Extraction, High-Throughput Sequencing, and Bioinformatics Analysis

Fourth-instar larvae in Section 2.3 were used for DNA extraction. The methods of larval surface disinfection and midgut tissue dissection are explained in Section 2.1. For each sample, 50 midguts were collected in a sterile centrifuge tube. The MN NucleoSpin 96 Soil DNA kit was used to extract total DNA from all samples. A NanoDrop 2000 spectrophotometer was used to measure the quantity and quality of total DNA. The total DNA was preserved at −80 °C. Each treatment had three replicates.

The V3-V4 hypervariable regions of the bacterial 16s rDNA gene were amplified with a universal primer containing sequencing adapter at the end of 338F (5′-ACTCCTACGGGAGGCAGCA-3′) and 806R (5′-GGACTACHVGGGTWTCTAAT-3′). First-round tailed polymerase chain reaction (PCR) amplification was performed in a volume of 10 µL with the following reaction components: 1 μL of the 10 μM primer F, 0.3 μL of the 10 μM primer R, 50 ng of genome DNA, 5 μL of KOD FX Neo Buffer, 2 μL of dNTP, and 0.2 μL of KOD FX Neo. PCR cycling parameters were 95 °C for 5 min, followed by 25 amplification cycles of 95 °C for 30 s, 50 °C for 30 s, and 72 °C for 40 s, with a 7 min final extension at 72 °C. The 2nd-round tailed PCR amplification was performed to add indices and adapter sequences in a volume of 20 μL comprising 10 μL of 2 × Q5 HF MM, 2.5 μL of MPPI-a, 2.5 μL of MPPI-b, and 5 μL of the products of 1st-round PCR. The reaction conditions are as follows: 98 °C for 30 s, followed by 10 cycles of 98 °C for 10 s, 65 °C for 30 s, 72 °C for 30 s, and a 5 min final extension at 72 °C. The PCR products were determined on 1.8% agarose gels and purified, quantified, and homogenized to form a sequencing library. Qualified libraries were sequenced using Illumina HiSeq 2500 by Biomarker Co., Ltd. (Beijing, China).

High-throughput sequencing generated a total of 480,468 paired-end reads. First, raw data were analyzed by the Trimmomatic software (version 0.33, Golm, Germany). Then, primer sequence recognition and removal were performed for high-quality reads that did not involve primer sequences by using cutadapt software (version 1.9.1, TU Dortmund, Germany). The clean reads were obtained by overlapping and splicing high-quality reads of each sample using FLASH software (version 1.2.7, Baltimore, MD, USA). The chimera sequences were identified and removed by UCHIME software (version 4.2, http://drive5.com/usearch/manual/uchime_algo.html accessed on 20 September 2021), resulting in effective reads. Operational taxonomic units (OTUs) were divided by USEARCH software (version 10.0, http://drive5.com/usearch/ accessed on 20 September 2021) based on 97% sequence similarity, and filtered at 0.005% of the total number of sequences. The representative sequences of each OTU, selected by the QIIME 2 software package, were annotated by the RDP Classifier software (https://sourceforge.net/projects/rdpclassifier/ accessed on 20 September 2021) at an 80% confidence threshold and blasted against the Silva 128 database (http://www.arb-silva.de accessed on 20 September 2021). Here, the biomarkers that are statistically different between treatment and control groups were screened by linear discriminant analysis (LDA) according to LDA scores greater than 3.5. The biomarkers distributed at different taxonomic levels were shown in a cladogram drawn online on the Galaxy. PICRUSt2 was used to annotate pathways of OTUs in the Cluster of Orthologous Groups (COG) database to predict microbiota functions.

### 2.5. Effects of Antibiotic Treatment on the Growth, Development, and Reproduction of G. molesta

Similar to Section 2.3, additional larvae were fed an artificial diet containing 1 μg/mL of ciprofloxacin for one generation until the next generation hatched to explore the effects of intestinal flora on *G. molesta* growing development. The artificial diet without antibiotics was used as a control. The developmental period of each larva was recorded at 9:00, 15:00, and 21:00 every day. A single mature larva was placed into a test tube (15 mm × 80 mm) for pupariation after it was out of feed and no longer fed, and the pupation period was recorded. After 48 h of pupation, the weight of a single pupa was measured with an analytical balance. There were three replicates in each treatment, and each replicate contained 100 newly hatched larvae. In order to prove the effects of gut microbiota on the longevity and fecundity of adults, male and female *G. molesta* that had just emerged were placed in pairs in a transparent plastic cup. A cotton ball dipped in 10% honey water was placed as a supplement for the adults. The cup was covered with plastic wrap, and some air holes were made with an insect needle. After numbering, it was placed in an artificial climate box for feeding. At 9:00, 15:00, and 21:00 every day, the number of eggs on the plastic film and the cup wall and the survival of the adults were observed and counted. If necessary (if there was no place on the inside of the plastic cup to lay eggs), the plastic cup was replaced until all the adults died, and the time of death was recorded. The hatching of eggs was observed and recorded, and the hatching rate of eggs was counted.

### 2.6. Isolation and Purification of Culturable Bacteria from the G. molesta Midgut

The culturable bacteria from the *G. molesta* 4th-instar larvae midgut fed an artificial diet within and without 1 μg/mL of ciprofloxacin was isolated by a traditional culture-dependent method. For each sample, 20 midguts were loaded into a 1.5 mL sterile centrifuge tube. After homogenization, the bacterial liquid was diluted to 10^−4^, 10^−5^, and 10^−6^. Then, a 50 μL dilution was drawn and spread on Luria–Bertani (LB) agar plates (tryptone 10 g/L, yeast extract 5 g/L, NaCl 10 g/L, and agar 15 g/L). The plates were incubated at 37 °C for 72 h. Colony differentiation in size, color, and morphology was observed every 24 h. Single representative isolates of each morphotype were transferred to new plates for purification 3 to 4 times by streak plate. Then, each purified isolate was identified by PCR amplification of the 16S rRNA gene using the universal bacterial forward primer 27F (5′-AGAGTTTGATCCTGGCTCAG-3′) and the reverse primer 1492R (5′-TACGGCTACCTTGTTACGACTT-3′). The 25 μL system (mix 12.5 μL, forward and reverse primers 1 μL each, plate 1 μL, and ddH_2_O 9.5 μL) was carried out with the following protocol: 94 °C for 3 min, 30 cycles at 94 °C for 30 s, 55 °C for 30 s, 72 °C for 30 s, and a final extension at 72 °C for 7 min. Amplification products were examined by electrophoresis in 1% agarose gels. Subsequently, PCR products were sequenced by bidirectional Sanger sequencing. The 16S rRNA sequence of each isolate was compared and aligned against cataloged sequences on the NCBI website.

We also identified culturable cellulase-producing bacteria in the gut of *G. molesta* before and after antibiotic treatment by a cellulase screening plate (sodium carboxymethylcellulose (CMC-Na) 10 g/L, tryptone 1 g/L, NaCl 1 g/L, K_2_HPO_4_ 2 g/L, MgSO_4_∙7H_2_O 0.4 g/L, agar 20 g/L, pH = 7.0) and a cellulase identification plate (CMC-Na 5 g/L, (NH_4_)_2_SO_4_ 2 g/L, K_2_HPO_4_ 2 g/L, NaCl 1 g/L, MgSO_4_∙7H_2_O 0.2 g/L, CaCl_2_ 0.1 g/L, agar 20 g/L, pH = 7.0). Except for the culture temperature set to 30 °C the other operations were as described above. The bacteria screened by the cellulase screening plate were inoculated on the cellulase identification plate and then cultured at 30 °C for five days, with *Escherichia coli* DH5-α as the control. Cellulase-producing bacteria were identified by the transparent zone assaying method. Appropriate 0.1% Congo red was added to the cellulase identification plate for 10 min and decolorized with 1 mol/L NaCl for 5 min to observe whether a transparent circle was generated around the colony (red background).

### 2.7. Bacteria Recolonization

The newly hatched larvae were fed with an antibiotic-containing diet until the 3rd instar, and the normal diet was the blank control. In order to recolonize the bacteria in the gut of the larvae, the 3rd-instar larvae were fed with the feed containing the bacterial solution for two hours after six hours of starvation, and the feed without the bacterial solution was set as the control. Then, the feed was transferred to the normal diet and fed to the pre-pupal stage, and the developmental stages of the larvae were compared.

## 3. Results

### 3.1. Evaluation of Antimicrobial Effects of the Different Antibiotics

**Figure 1:**
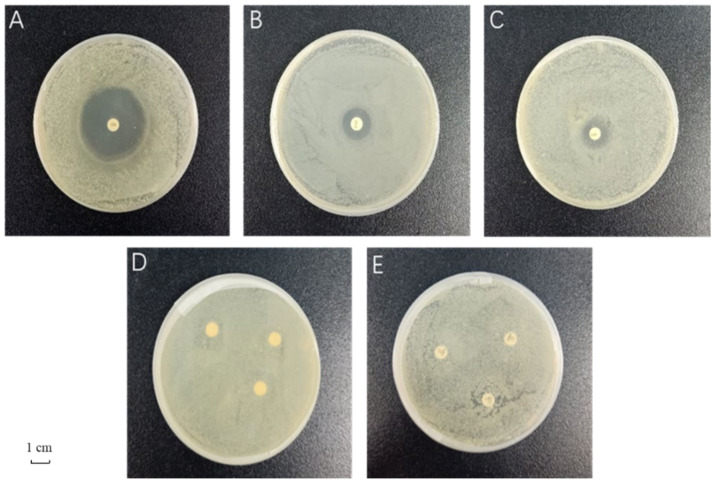
Bacteriostatic effect of five antibiotics on gut bacteria of *Grapholita molesta*. (**A**) ciprofloxacin; (**B**) streptomycin; (**C**) chloramphenicol; (**D**) rifampicin; (**E**) ampicillin.

The sensitivity evaluation results of antibiotics are shown in Table 1 and Figure 1. Of the five antibiotics, ciprofloxacin and streptomycin were sensitive, chloramphenicol was moderately sensitive, and rifampicin and ampicillin were resistant. Among the two sensitive antibiotics, ciprofloxacin had the largest inhibition zone diameter (37.7 ± 0.6 mm), which was significantly higher than that of streptomycin (23.3 ± 1.5 mm). Therefore, ciprofloxacin was selected as the most suitable antibiotic to inhibit the gut microbiota of *G. molesta.*

### 3.2. Screening of the Minimum Inhibitory Concentration

**Figure 2:**
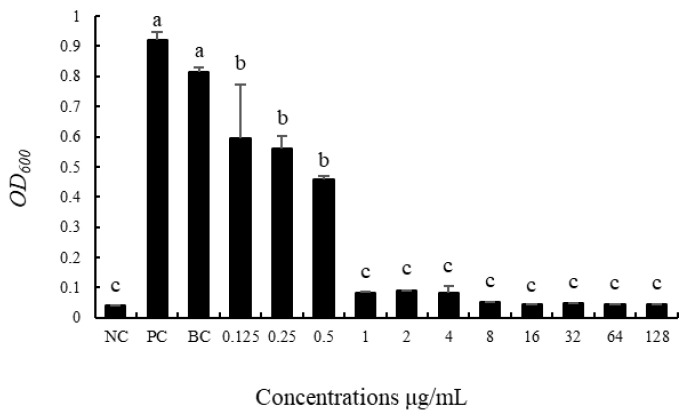
Antibacterial effects of different concentrations of ciprofloxacin on cultivable gut microbiota of *Grapholita molesta*. NC: negative control; PC: positive control; BC: blank control. The different lowercase letters indicate significant differences among different treatments (one-way ANOVA, Tukey post-hoc test, *p* < 0.05).

The broth method was used for measurement, and the *OD*_600_ value was measured after 24 h culture at 37 °C, as shown in Figure 2. The bacteria in the positive control group with only the addition of the sample bacterial solution grew significantly, and the *OD*_600_ value was 0.9206 ± 0.0252. The bacteria in the blank control group also grew significantly, and the *OD*_600_ value was 0.8161 ± 0.0127. There was no significant difference between the two groups. The *OD*_600_ value of the treated group was significantly lower than that of the positive and blank control groups, indicating that ddH_2_O dissolution did not affect the experiment, and the antibacterial effect was caused by ciprofloxacin. It can be seen from Figure 2 that ciprofloxacin at the concentration of 1 μg/mL completely inhibited the growth in vitro of the cultivable intestinal bacteria of *G. molesta*, and the *OD*_600_ value did not change significantly with an increase in the concentration. Ciprofloxacin at the concentrations of 0.125 μg/mL, 0.25 μg/mL, and 0.5 μg/mL also had certain bacteriostatic effects. There were no significant differences in the *OD*_600_ value among the three concentrations, but they were significantly higher than the *OD*_600_ value of 1 μg/mL; that is, the bacteriostatic effect was significantly lower than 1 μg/mL. According to the relevant standards of CLSI (2012), the minimum inhibitory concentration of ciprofloxacin against cultivable intestinal bacteria of *G. molesta* was determined to be 1 μg/mL, and subsequent experiments were conducted under this concentration.

### 3.3. Effects of Ciprofloxacin on the Gut Microbiota Structure of G. molesta

A total of 480,468 pairs of raw reads were obtained from six samples before and after antibiotic treatment by Illumina HiSeq 2500 sequencing, and a total of 478,791 clean reads were generated after double-ended read quality control and splicing. As shown in Table 2, a single sample produced a minimum of 79,536 clean reads, a maximum of 80,209 clean reads, and an average of 79,799 clean reads. Q20 was above 99%, Q30 was above 96%, and the proportion of valid data obtained after removal of chimeras was above 95%, indicating good sequencing quality. Reads were clustered with USEARCH software at a similarity level of 97.0%, and 1980 OTUs were obtained, including 28 phyla, 55 classes, 134 orders, 242 families, 542 genera, and 832 species.

**Figure 3:**
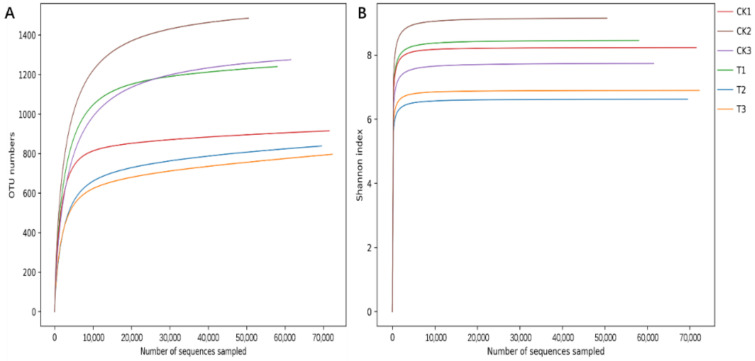
Rarefaction curve and Shannon index rarefaction curve of *Grapholita molesta* gut microbiota samples before and after ciprofloxacin feeding. (**A**) Rarefaction curve; (**B**) Shannon index rarefaction curve.

The sample rarefaction curve (Figure 3) shows that the number of OTUs increases sharply with the increase in sample sequencing quantity at first, and then the slope gradually decreases when it reaches a certain value, and finally gradually flattens out and enters the plateau stage, indicating that continued increase in sequencing quantity can only produce a small number of OTUs. Therefore, the sequencing depth at this time has basically covered all bacterial species in the sample, and more sequencing would contribute little to the generation of new OTUs. The Shannon index rarefaction curve showed that the Shannon index of all samples increased sharply at first, then rapidly leveled off and entered a plateau, indicating that the sequencing depth was sufficient to cover most of the microbial information in the samples. At the same time, the α diversity of the sequencing samples was analyzed. Compared with the control group, the ACE, Chao 1, Shannon, and Simpson indices of intestinal microflora were all decreased after feeding antibiotics, such as ciprofloxacin, but the differences were not significant. In addition, the coverage rate of all samples was greater than 0.99, indicating that the real situation of the microorganisms could be reflected (Table 3).

**Figure 4:**
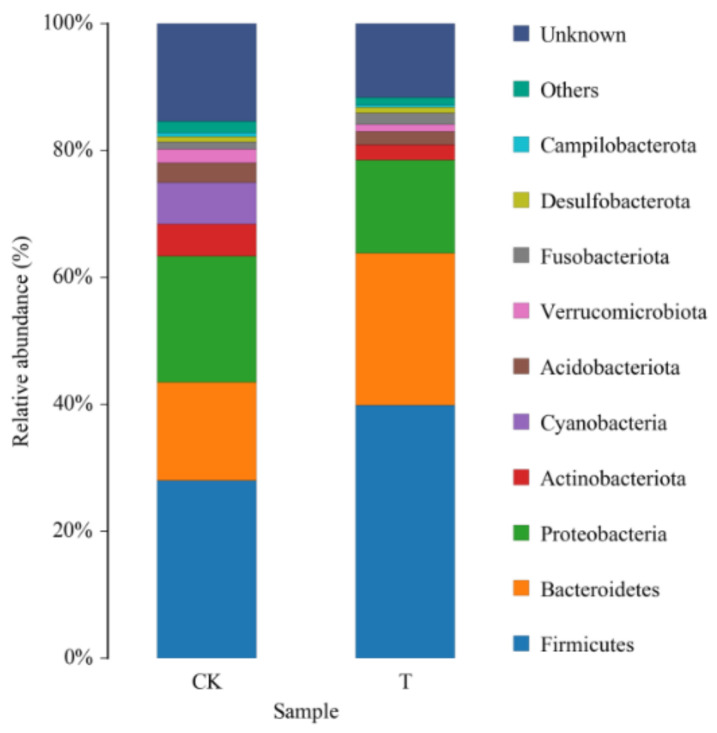
Histogram of the relative abundance of top 10 phyla of the gut microbiota samples of *Grapholita molesta* before and after antibiotic feeding. CK: control; T: treatment with ciprofloxacin. Each color represents a species, and the height of the color block indicates the proportion of the species in relative abundance. Other species are incorporated as “Others” shown in the diagram. “Unknown” represents species that have not received a taxonomic annotation.

The histogram of relative abundance in species distribution in the top 10 phyla levels is shown in Figure 4. At the phylum level, the composition of dominant bacteria in the *G. molesta* gut was similar before and after feeding antibiotics, but the relative abundance of different bacteria changed. After feeding ciprofloxacin, the relative abundance of Actinobacteria and Cyanobacteria decreased significantly, while that of Firmicutes and Bacteroidetes increased from 27.40 ± 4.99% and 15.33 ± 1.64% to 39.56 ± 3.15% and 23.67 ± 2.54%, respectively (Table 4). These results indicate that there are a large number of ciprofloxacin antibiotic-resistant bacteria in Firmicutes and Bacteroidetes, which may be related to the resistance of *G. molesta*.

**Figure 5:**
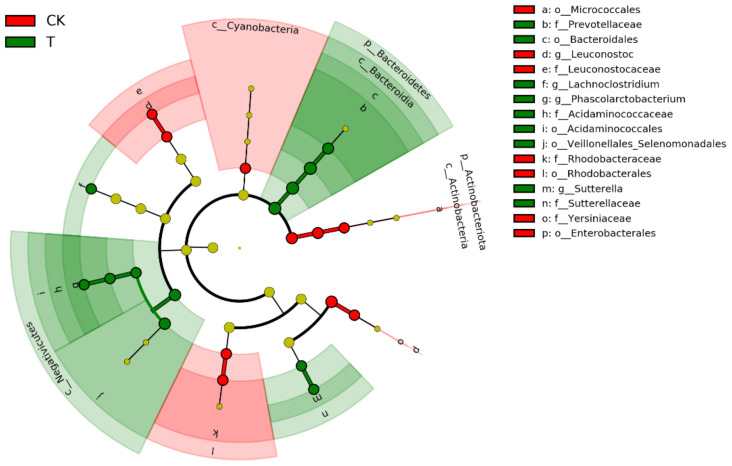
Cladogram of bacterial biomarkers of the gut microbiota samples of *Grapholita molesta*, from the phylum (innermost ring) to genus (outermost ring) level, with an LDA score > 3.5. CK: control; T: treatment with ciprofloxacin. Each small circle represents a taxon, and a large diameter of the circle indicates a high relative abundance of that taxon. Different lowercase letters represent different bacterial taxa. Taxa with no insignificant differences are shown in yellow, taxa with higher relative abundance in treatment groups than control groups are shown in green, and taxa with higher relative abundance in control groups than treatment groups are shown in red.

There are 12 taxa mainly concentrated in Bacteroidetes, Firmicutes, and Proteobacteria in the gut microbiota of *G. molesta* after treatment with ciprofloxacin, especially in Negativicutes. In contrast, the 10 taxa in the control group were mainly distributed in Cyanobacteria, Actinobacteria, and Proteobacteria, and fewer in Bacillibacteria of Firmicutes. Overall, these analyses confirmed that ciprofloxacin supplementation alters the intestinal flora of *G. molesta* (Figure 5).

**Figure 6:**
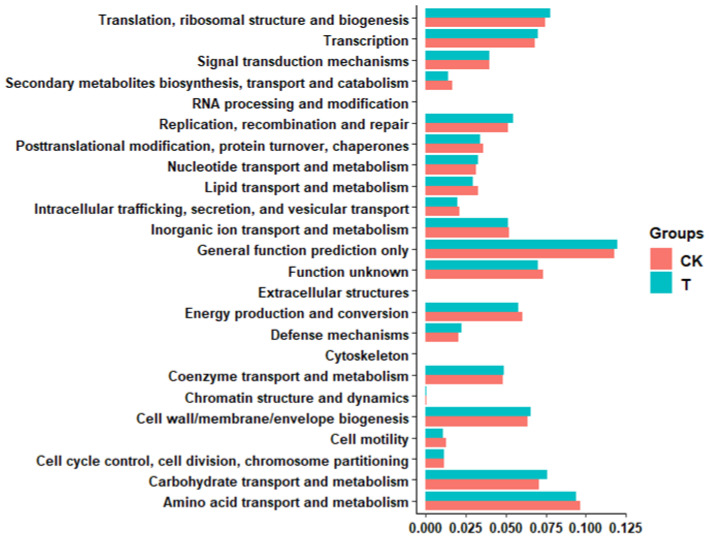
Comparison of predicted Cluster of Ortholog Groups (COG) functions of gut bacteria of *Grapholita molesta* before and after ciprofloxacin feeding. The X-axis represents the abundance value of functional categories, and the Y-axis represents the different function categories. CK: control; T: treatment with ciprofloxacin.

In order to better illustrate the important part of the gut microbiota of *G. molesta*, PICRUSt2 software was used to predict the functional gene compositions of samples based on 16S rDNA sequencing data and compared them with the Cluster of Orthologous Groups (COG) database. The results indicated that most functional prediction categories, including amino acid transport and metabolism, translation, ribosomal structure and biogenesis, carbohydrate transport and metabolism, transcription, cell wall/membrane/envelope biogenesis, energy production, and conversion, were enriched in the gut of *G. molesta*. And the differences in the function of *G. molesta* intestinal flora before and after ciprofloxacin feeding were noticed in the functions of carbohydrate transport and metabolism, cell wall/membrane/envelope biogenesis, replication, recombination and repair, etc (Figure 6).

### 3.4. Effects of Antibiotic Treatment on the Growth, Development, and Reproduction of G. molesta

The developmental stages of the *G. molesta* larvae before and after feeding 1 μg/mL of ciprofloxacin are shown in Table 5. The larval stage of *G. molesta* after treatment was observably longer than the control group, reaching 14.47 ± 0.20 d, 0.93 d longer than that of the control group. The adult longevity was shortened by 1.18 d, which was significantly different from that of the control group. However, there was no distinct difference between the treatment group and the control group in the pupal stage and the total duration from larva to adult. In general, treatment with 1 μg/mL of ciprofloxacin mainly affected the development of the larval stage and the adult stage, resulting in a prolonged larval stage and significantly shortened adult longevity.

Biological parameters of *G. molesta* before and after feeding 1 μg/mL of ciprofloxacin at different developmental stages were recorded (Table 6). The larval survival rate, pupation rate, pupal weight, and emergence rate of *G. molesta* in the treatment group were decreased, but the difference was not significant compared with the control group. The number of eggs laid by a single female in the control group was 122.13 ± 4.96, but that in the treatment group was 87.54 ± 5.72 after 1 μg/mL of ciprofloxacin feeding, which was significantly lower than the control group. Similarly, the egg hatching rate was significantly decreased, 80.20 ± 3.30% in the control group and 58.24 ± 6.69% in the treatment group. In general, antibiotic supplementation with 1 μg/mL of ciprofloxacin significantly affected the larval development duration, adult longevity, single female oviposition, and egg hatchability, resulting in prolonged larval development duration, shortened adult longevity, and significantly decreased single female oviposition and, egg hatchability.

### 3.5. Identification of Culturable Gut Bacteria

Identical with the results of 16S amplicon sequencing, the results of the in vitro culture experiment were different before and after antibiotic treatment. We isolated and cultured 10 and 5 species from the control and treatment groups, respectively. Most of the bacteria isolated from the cultures belonged to Proteobacteria, Firmicutes, and Actinobacteria (Table 7).

**Figure 7:**
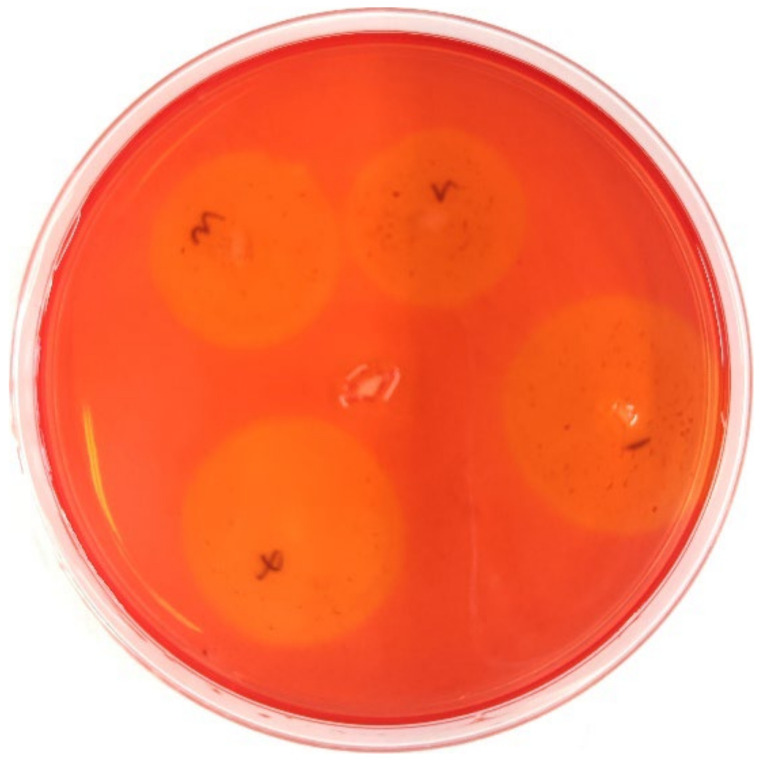
Transparent circles formed by cellulase-producing bacteria isolated from the control group. Around the plate are cellulase-producing bacteria and in the middle are *Escherichia coli* DH5-α controls.

For cellulase-producing bacteria, transparent zone assaying showed that four strains of bacteria formed clear circles in the control group, but *Escherichia coli* did not (Figure 7). Amplification of 16S sequencing revealed that only three strains, *Sphingobacterium* species., *Stenotrophomonas* species, and uncultured *Sphingobacterium*, were identified with identity values of more than 99%.

### 3.6. Effects of Cellulase-Producing Bacteria on the Growth and Development of G. molesta Larvae

The development time of *G. molesta* in the larval stage after feeding the isolated cellulase-producing bacteria compared to antibiotic-treated *G. molesta* is shown in Table 8. Similar to the previous results, the development duration of the larvae was significantly prolonged after antibiotic treatment. However, cellulase-producing bacteria had no significant effect on the development duration of the larvae treated with antibiotics, contrary to our expectations.

## 4. Discussion

In order to screen out antibiotics with better inhibitory effects on gut microbiota of *G. molesta*, five broad-spectrum antibiotics were selected for sensitivity testing in this experiment. The results demonstrated that the intestinal bacteria of *G. molesta* showed different sensitivities to different antibiotics: the intestinal bacteria of *G. molesta* were sensitive to ciprofloxacin and streptomycin, moderately sensitive to chloramphenicol, and resistant to rifampicin and ampicillin. The results were similar to those for the sensitivity of *Anticarsia gemmatalis* gut bacteria to different antibiotics [41]. There were significant differences in antibacterial zone diameter, and ciprofloxacin had the largest antibacterial zone diameter. The minimum inhibitory concentration was 1 μg/mL by using the microbroth method. Similar to the research that ciprofloxacin and piperacillin played the most important role in inhibiting bacterial growth of intestinal bacteria of *Ceratitis capitata* through sensitivity testing. However, the minimum inhibitory concentration of ciprofloxacin against the intestinal bacteria of *C. capitata* was 5 μg/mL, which was much higher than the 1 μg/mL in this study, indicating that the intestinal bacteria of *G. molesta* were more sensitive to ciprofloxacin [35]. Ciprofloxacin is a broad-spectrum antibiotic, belonging to the fluoroquinolone antibiotics. Norfloxacin, also a fluoroquinolone, had a good inhibitory effect on *Eurygaster integriceps* intestinal bacteria [42].

After feeding ciprofloxacin, the diversity of intestinal microbiota of *G. molesta* decreased. Although the differences were not significant, the ACE, Chao 1, Shannon, and Simpson indices all showed varying degrees of reduction. Perhaps this is due to the fact that this experiment only raised and studied one generation, a length of time too short for antibiotic action. Another reason may be that the antibiotic types screened in vitro only inhibit the culturable bacteria in the *G. molesta* gut, while the inhibitory effect on the unculturable bacteria is unknown. In addition, the antibiotic solution directly acts on the bacterial solution in the in vitro screening concentration experiments, while *G. molesta* was fed with antibiotics through the digestive tract to the midgut; this process may also reduce the antibacterial effect of the antibiotics. Similar results have been found in previous studies. For example, the richness and diversity of gut microbiota of the small brown planthopper decreased after feeding it with 1 g/L of tetracycline for five generations, but the difference was not significant [25]. When mixed solutions of four different concentrations of three antibiotics (tetracycline, ampicillin, and streptomycin) were used to interfere with the intestinal microbes of *Bactrocera dorsalis* reared in the laboratory, no prominent differences were observed in the bacterial diversity before and after treatment [40]. The richness and diversity of the intestinal microbiota of *Z. tau* larvae had no significant variation after being fed with three different concentrations of antibiotic solutions (ampicillin, streptomycin sulfate, and tetracycline) to the third instar; however, *Enterobacter*, *Providencia*, *Serratia*, and *Klebsiella* were almost completely eliminated after treatment, and *Enterococcus*, which had low abundance, became the new dominant bacteria after treatment [36]. In this study, after treatment with ciprofloxacin, there was no increase of the non-dominant bacteria to the dominant bacteria in the intestinal microbiota of *G. molesta*, but only changes in the relative abundance of the dominant bacteria. At the phylum level, the relative abundance of Proteobacteria, Actinomycetes, and Cyanobacteria decreased, while the variations of the latter two phyla were significant. Interestingly, after feeding antibiotics, the relative abundance of Firmicutes and Bacteroidetes increased from 27.40 ± 4.99% and 15.33 ± 1.64% to 39.56 ± 3.15%, and 23.67 ± 2.54%, respectively. These results imply that a large number of ciprofloxacin-resistant bacteria consist of Firmicutes and Bacteroidetes, which could serve as an entry point for subsequent studies of *G. molesta* resistance.

Many studies have reported that symbiotic bacteria play a key role in insect growth and development [13,31,42,43,44,45,46,47,48]. In our study, larval development duration was significantly prolonged, and the adult development duration was significantly shortened after treatment with 1 μg/mL of ciprofloxacin, suggesting a potential fitness advantage for *G. molesta* intestinal bacteria. Similar results have been found in experiments with antibiotic feeding of *Pieris canidia*. After being fed with tetracycline at three different concentrations, the larval and pupal stages were significantly prolonged, and the adult stage was significantly shortened. With an increase in antibiotic concentration, the influence on the development period of *P. canidia* was more significant [49].

The gut microbiota not only has an effect on the growth and development of the host, but also plays an important role in the reproduction of the host, indicating that the intimate association between insects and their gut microbiota extends beyond nutritional support to behavior [50]. It has been shown that untreated flies of *Bactrocera oleae* that had native gut symbionts attempted oviposition significantly more times than axenic flies [50]. The ovaries of the female adults did not develop or lay eggs 20 days after eclosion after feeding the *Z. tau* larvae with three different concentrations of antibiotics, while the control group normally laid eggs [36]. The number of eggs laid by an adult female *B. dorsalis* was significantly higher than control after adding *Pantoea dispersa* and *Enterobacter cloacae* into the feed [51]. In this study, it was found that the single female oviposition and egg hatching rates of *G. molesta* were significantly reduced after treatment with 1 μg/mL of ciprofloxacin, suggesting that intestinal bacteria could help *G. molesta* produce more fertile eggs. The mechanisms by which gut symbionts modulate host physiologies and the molecules involved in these changes have been reported as follows: *Burkholderia* gut symbiont modulates *Riptortus pedestris* development and egg production by regulating production of three hemolymph storage proteins encoded by hexamerin-α, hexamerin-β, and vitellogenin-1, respectively [31]. It has also been reported that the gut symbiotic bacteria *Enterococcus* species can improve the oviposition repellence and fitness of *Drosophila* by producing a large number of organic acids in the fermentation process to neutralize the alkaline environment of the oviposition matrix [52].

Cases of antibiotic resistance have been increasing over the past few decades. Horizontal gene transfer between pathogens and insect gut commensal microorganisms has been revealed [53]. Although it has been revealed that the H_2_S-mediated signal path is associated with bacterial tolerance, biofilm, and virulence, providing a solution to antibiotic resistance [54]. We still think it is feasible to use antibiotics to investigate the function of microorganisms in the laboratory, but we should be cautious about applying this approach to the wild.

## 5. Conclusions

Our results demonstrated that the gut bacterial structure of *G. molesta* can be influenced by ciprofloxacin with a minimum inhibitory concentration of 1 μg/mL. After ciprofloxacin treatment, the relative abundance of Actinobacteria and Cyanobacteria decreased significantly, while that of Firmicutes and Bacteroidetes increased. PICRUSt2 analysis indicated that most functional prediction categories, including amino acid transport and metabolism, translation, ribosomal structure and biogenesis, carbohydrate transport and metabolism, transcription, cell wall/membrane/envelope biogenesis, and energy production and conversion, were enriched in the gut of *G. molesta*. Finally, ciprofloxacin feeding significantly affected larval growth, development, and reproduction, resulting in prolonged larval development duration, shortened adult longevity, and significantly decreased single female oviposition and egg hatchability. In addition, we isolated and purified some culturable bacteria and cellulase-producing bacteria from the *G. molesta* midgut. In brief, our results confirmed that gut bacterial communities of *G. molesta* can be influenced by antibiotics, which is beneficial for host growth, development, and reproduction. These results will provide a theoretical reference for developing new *G. molesta* control targets and green control technologies.

## Figures and Tables

**Table 1:** Inhibition zone diameters of five antibiotics on the growth of culturable gut microbiota of *Grapholita molesta* at MH agar plate.

Antibiotic	Content/μg	Diameter of Bacteriostatic Zone/mm	Criteria for Determination of Bacteriostatic Effect/mm			Result
			Resistant	Moderate	Sensitive	
Ciprofloxacin	5	37.7 ± 0.6 a	≤15	16–20	≥21	Sensitive
Streptomycin	10	23.3 ± 2.1 b	≤11	12–14	≥15	Sensitive
Chloramphenicol	30	16.0 ± 1.5 c	≤12	13–17	≥18	Moderate
Rifampicin	5	0 ± 0.0 d	≤16	17–19	≥20	Resistant
Ampicillin	10	0 ± 0.0 d	≤13	14–16	≥17	Resistant

Data in the table are means ± SEM. The different lowercase letters in the third column indicate significant differences among different treatments (one-way ANOVA, Tukey post-hoc test, *p* < 0.05).

**Table 2:** Statistical results of the sequencing data of *Grapholita molesta* gut microbiota before and after ciprofloxacin feeding.

Sample ID	Raw Reads	Clean Reads	Effective Reads	AvgLen (bp)	GC(%)	Q20(%)	Q30(%)	Effective (%)
CK1	80,066	79,771	76,755	417	53.50	99.20	96.67	95.86
CK2	80,265	80,017	76,925	417	54.61	99.20	96.68	95.84
CK3	79,983	79,662	76,948	417	54.09	99.17	96.57	96.21
T1	80,452	80,209	77,087	417	53.83	99.15	96.52	95.82
T2	79,820	79,536	76,753	419	52.79	99.14	96.45	96.16
T3	79,882	79,596	77,027	417	52.25	99.15	96.50	96.43

CK: control; T: treatment with ciprofloxacin. GC: The ratio of guanine (G) + cytosine (C) in sequences. Q20: The percentage of bases with a mass value ≥ 20. Q30: The percentage of bases with a mass value ≥ 30.

**Table 3:** Statistical analysis of α diversity index of *Grapholita molesta* gut microbiota before and after ciprofloxacin feeding.

Sample ID	ACE	Chao 1	Shannon	Simpson	Coverage
CK	1269.3823 ± 156.1559 a	1301.3513 ± 147.2708 a	8.3728 ± 0.4138 a	0.9859 ± 0.0073 a	0.9986 ± 0.0003 a
T	1091.6186 ± 98.2405 a	1150.1026 ± 104.2863 a	7.3237 ± 0.5705 a	0.9740 ± 0.0081 a	0.9985 ± 0.0001 a

CK: control; T: treatment with ciprofloxacin. Data in the table are means ± SEM. The different lowercase letters in the same column indicate significant differences between different treatments (one-way ANOVA, Tukey post-hoc test, *p* < 0.05).

**Table 4:** Changes in the relative abundance of top 10 phyla in *Grapholita molesta* gut microbiota samples before and after ciprofloxacin feeding.

Group	Firmicutes	Bacteroidetes	Proteobacteria	Actinobacteriota	Cyanobacteria	Acidobacteriota	Verrucomicrobiota	Fusobacteriota	Desulfobacterota	Campilobacterota
CK	27.40 ± 4.99 a	15.33 ± 1.64 a	20.17 ± 2.07 a	5.20 ± 0.48 a	9.68 ± 0.19 a	3.38 ± 1.24 a	2.21 ± 0.49 a	1.13 ± 0.38 a	0.82 ± 0.11 a	0.64 ± 0.10 a
T	39.56 ± 3.15 a	23.67 ± 2.54 a	14.89 ± 1.89 a	2.42 ± 0.38 b	0.07 ± 0.03 b	2.19 ± 0.95 a	1.21 ± 0.73 a	1.72 ± 1.19 a	0.97 ± 0.22 a	0.31 ± 0.12 a

CK: control; T: treatment with ciprofloxacin. Data in the table are means ± SEM. The different lowercase letters in the same column indicate significant differences between different treatments (one-way ANOVA, Tukey post-hoc test, *p* < 0.05).

**Table 5:** Development duration of *Grapholita molesta* after antibiotic feeding.

Group	Larval Duration	Pupal Duration	Adult Longevity	Larval-Adult Total Duration
CK	13.54 ± 0.04 b	7.11 ± 0.12 a	17.86 ± 0.16 a	38.11 ± 0.06 a
T	14.47 ± 0.20 a	7.24 ± 0.03 a	16.68 ± 0.36 b	38.11 ± 0.92 a

CK: control; T: treatment with ciprofloxacin. Data in the table are means ± SEM. The different lowercase letters in the same column indicate significant differences between different treatments (one-way ANOVA, Tukey post-hoc test, *p* < 0.05).

**Table 6:** Effects of antibiotics on growth, development, and reproduction of *Grapholita molesta*.

Group	CK	T
Larval survival rate/%	50.67 ± 1.45 a	48.33 ± 2.03 a
Pupation rate/%	94.13 ± 0.98 a	92.34 ± 2.00 a
Pupal weight/mg	10.78 ± 0.27 a	10.55 ± 0.22 a
Emergence rate/%	87.38 ± 2.46 a	86.49 ± 0.76 a
No. eggs per female	122.13 ± 4.96 a	87.54 ± 5.72 b
Egg hatchability/%	80.20 ± 3.30 a	58.24 ± 6.69 b

CK: control; **T**: treatment with ciprofloxacin. Data in the table are means ± SEM. The different lowercase letters in the same row indicate significant differences between the two treatments (one-way ANOVA, Tukey post-hoc test, *p* < 0.05).

**Table 7:** NCBI Blast results of 16S sequence of culturable bacteria from *Grapholita molesta*.

Group	Clone No.	Origin Strain of Blasted Sequences with the Highest Identity	Phylum	GenBank Accession No.	Identity (%)
CK	CK1	*Staphylococcus warneri*	Firmicutes	MH910124.1	100
	CK2	*Micrococcus* sp.	Actinobacteria	LC484692.1	99.93
	CK3	*Microbacterium imperiale*	Actinobacteria	JN585685.1	100
	CK4	*Microbacterium arborescens*	Actinobacteria	MN198184.1	100
	CK5	*Staphylococcus* sp.	Firmicutes	MK757916.1	99.86
	CK6	*Staphylococcus epidermidis*	Firmicutes	MW391759.1	99.93
	CK7	*Staphylococcus hominis* subsp. *hominis*	Firmicutes	KX510094.1	99.93
	CK8	*Staphylococcus hominis*	Firmicutes	MW391758.1	99.93
	CK9	*Staphylococcus hominis* subsp. *novobiosepticus*	Firmicutes	MT544719.1	99.86
	CK10	*Microbacterium oxydans*	Actinobacteria	KF358264.1	100
T	T1	*Pantoea septica*	Proteobacteria	ON197340.1	99.44
	T2	*Kitasatospora* sp. strain C15	Actinobacteria	MG461679.1	99.58
	T3	*Niallia circulans* strain S1	Firmicutes	MK100762.1	99.93
	T4	*Staphylococcus hominis subsp. novobiosepticus* strain 4149	Firmicutes	MT544719.1	99.93
	T5	*Pantoea* sp. strain GIg21	Proteobacteria	MK312599.1	100

CK: control; T: treatment with ciprofloxacin.

**Table 8:** Larval development duration after recolonization of cellulase-producing bacteria.

Group	CK	T
BC	13.93 ± 0.13 b	7.68 ± 0.03 b
CK	14.86 ± 0.12 a	8.62 ± 0.04 a
*Sphingobacterium* species	14.66 ± 0.10 a	8.49 ± 0.09 a
*Stenotrophomonas* species	14.86 ± 0.13 a	8.45 ± 0.16 a
uncultured *Sphingobacterium*	14.80 ± 0.09 a	8.54 ± 0.10 a

Data in the table are means ± SEM. The different lowercase letters in the same row indicate significant differences between different treatments (one-way ANOVA, Tukey post-hoc test, *p* < 0.05).

## Data Availability

The data that support this study is openly available in GenBank of NCBI at https://submit.ncbi.nlm.nih.gov/subs/. The associated BioProject accession number is PRJNA778521.
